# Lentivirus-mediated interleukin-1β (IL-1β) knock-down in the hippocampus alleviates lipopolysaccharide (LPS)-induced memory deficits and anxiety- and depression-like behaviors in mice

**DOI:** 10.1186/s12974-017-0964-9

**Published:** 2017-09-20

**Authors:** Mengmeng Li, Chenli Li, Hanjie Yu, Xiongxiong Cai, Xinbei Shen, Xin Sun, Jinting Wang, Yanhua Zhang, Chuang Wang

**Affiliations:** 1 Ningbo Key Laboratory of Behavioral Neuroscience, Ningbo University School of Medicine, 818 Fenghua Road, Ningbo, Zhejiang 315211 People’s Republic of China; 2 Zhejiang Provincial Key Laboratory of Pathophysiology, Ningbo University School of Medicine, 818 Fenghua Road, Ningbo, Zhejiang 315211 People’s Republic of China; 3 Department of Physiology and Pharmacology, Ningbo University School of Medicine, 818 Fenghua Road, Ningbo, Zhejiang 315211 People’s Republic of China; 4 Li Dak Sum Yip Yio Chin Kenneth Li Marine Biopharmaceutical Research Center, Ningbo University, Ningbo, 315211 People’s Republic of China

**Keywords:** Interleukin-1β (IL-1β), Lipopolysaccharides (LPS), Memory deficits, Depression, Anxiety

## Abstract

**Background:**

Recent evidence has suggested that peripheral inflammatory responses induced by lipopolysaccharides (LPS) play an important role in neuropsychiatric dysfunction in rodents. Interleukin-1β (IL-1β), a pro-inflammatory cytokine, has been proposed to be a key mediator in a variety of behavioral dysfunction induced by LPS in mice. Thus, inhibition of IL-1β may have a therapeutic benefit in the treatment of neuropsychiatric disorders. However, the precise underlying mechanism of knock-down of IL-1β in repairing behavioral changes by LPS remains unclear.

**Methods:**

The mice were treated with either IL-1β shRNA lentivirus or non-silencing shRNA control (NS shRNA) lentivirus by microinjection into the dentate gyrus (DG) regions of the hippocampus. After 7 days of recovery, LPS (1 mg/kg, i.p.) or saline was administered. The behavioral task for memory deficits was conducted in mice by the novel object recognition test (NORT), the anxiety-like behaviors were evaluated by the elevated zero maze (EZM), and the depression-like behaviors were examined by the sucrose preference test (SPT) and the forced swimming test (FST). Furthermore, the levels of malondialdehyde (MDA), superoxide dismutase (SOD), nuclear factor erythroid-derived 2-like 2 (Nrf2), heme oxygenase 1 (HO1), IL-1β, tumor necrosis factor (TNF-α), neuropeptide VGF (non-acronymic), and brain-derived neurotrophic factor (BDNF) were assayed.

**Results:**

Our results demonstrated that IL-1β knock-down in the hippocampus significantly attenuated the memory deficits and anxiety- and depression-like behaviors induced by LPS in mice. In addition, IL-1β knock-down ameliorated the oxidative and neuroinflammatory responses and abolished the downregulation of VGF and BDNF induced by LPS.

**Conclusions:**

Collectively, our findings suggest that IL-1β is necessary for the oxidative and neuroinflammatory responses produced by LPS and offers a novel drug target in the IL-1β/oxidative/neuroinflammatory/neurotrophic pathway for treating neuropsychiatric disorders that are closely associated with neuroinflammation, oxidative stress, and the downregulation of VGF and BDNF.

## Background

Growing evidence suggests a close link between neuroinflammation and neuropsychiatric disorders (e.g., memory deficits, depression, and anxiety) [1–4]. Acute treatment with the cytokine inducer lipopolysaccharide (LPS) is widely accepted in animal models to investigate the relationship between neuroinflammation and memory deficit, anxiety, and depression [2, 5, 6]. Indeed, behavioral dysfunction can be observed in LPS-stimulated animal models together with elevated pro-inflammatory cytokine levels and oxidative stress parameters in the hippocampus and other brain regions [7–10]. There is growing evidence that blocking the interleukin-1β (IL-1β) receptor is a therapeutic strategy for neuropsychiatric behavioral dysfunction in rodents [11, 12], indicating IL-1β as a key candidate for the treatment of neuropsychiatric disorders. IL-1β administration induces fatigue, anhedonia, weight loss, impaired social interaction, and memory deficits in animal models [13–15]. Therefore, insight into the factors affecting IL-1β may lead to novel strategies for establishing a treatment for neuropsychiatric disorders. However, the role of IL-1β in LPS-induced behavioral dysfunction and neurotrophic factor loss [7–10] has not been elucidated in mice. We evaluated whether IL-1β knock-down induced by lentivirus in the hippocampus shows memory enhancing, anti-depression, and anxiolytic effects in the background of acute neuroinflammation induced by LPS in mice.

Increasing evidence indicates that the neurotrophic hypothesis of neuropsychiatric disorders is based on the downregulation of brain-derived neurotrophic factor (BDNF) and the neuropeptide VGF (no acronym) expression in the brain [16–21]. In addition, previous studies have demonstrated that LPS-induced pro-inflammatory cytokine mediate the generation of oxidative parameters [9, 22] and the impairment of BDNF [23] and VGF functions [7]. Intervening in the process of neuroinflammation along with oxidative stress may be beneficial for the therapy of neuropsychiatric disorders. However, very few studies have assessed the relationship between IL-1β-mediated neuroinflammatory/oxidative signaling and BDNF or VGF in neuropsychiatric disorders. In our present work, we further assessed whether the possible protective effects of this IL-1β knock-down are associated with a decrease in pro-inflammatory cytokines and oxidative parameters, which are further implicated in the changes of BDNF and VGF in LPS-challenged mice.

## Methods

### Animals

Experiments were conducted on 8- to 10-week-old male imprinting control region (ICR) mice born and reared in the Zhejiang Academy of Medical Sciences, China. All animals were maintained at 22 ± 3 °C and 60% ± 5% relative humidity under a 12-h light/dark cycle (lights on at 7:00 a.m.) with ad libitum access to food and water. All animal experiments were conducted on animals outside of their housing area in a separate procedure room. All animal experiments were performed according to the National Institutes of Health (NIH) Guide for the Care and Use of Laboratory Animals (NIH Publication No. 80–23, revised 1996) and were approved by the Institutional Animal Care and Use Committee of the Medical School of Ningbo University.

In the present study, animals were divided into four experimental groups (*n* = 16 mice/group) for behavioral and biochemical assessments. Subsequent treatments were as follows: (1) Group 1 was treated with the non-silencing shRNA control (NS shRNA) lentivirus and saline. (2) Group 3 was treated with the IL-1β shRNA lentivirus and saline. (3) Group 2 was treated with the NS shRNA lentivirus and LPS (1.0 mg/kg, i.p.). (4) Group 4 was treated with the IL-1β shRNA lentivirus and LPS (1.0 mg/kg, i.p.). All behavioral testing were performed during the light period (between 8:00 and 17:00) under dim light. At the end of all treatments and behavioral tests, the mice were killed and the bilateral hippocampus (32 hippocampi per group) was removed for biochemical analyses.

### Construction of the IL-1β shRNA lentivirus

**Fig. 1:**
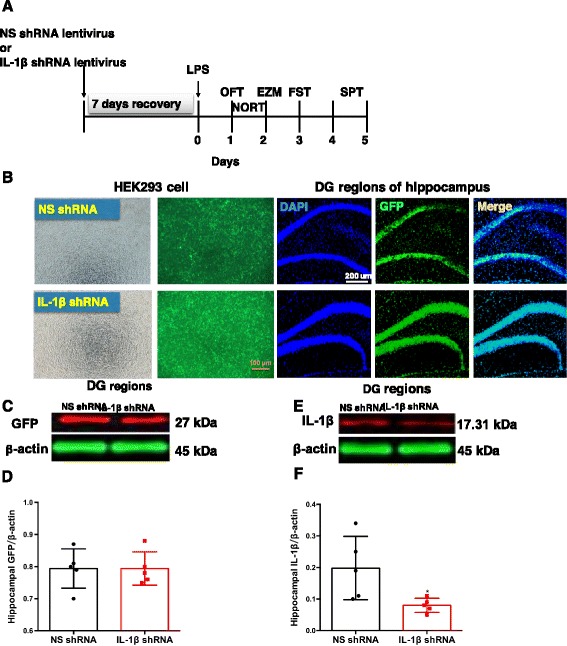
Timeline of the experimental design and confirmation of the effectiveness of the IL-1β shRNA lentivirus in mice. **a** Experimental procedure for the test schedule. NS shRNA or IL-1β shRNA were microinfused into bilateral DG regions of the hippocampus of mice, followed by a 7-day recovery. LPS (1 mg/kg, i.p.) or its vehicle was administered 7 days after the viral infusions (day 0), and then, 24 h later, the OFT, NORT, EZM, FST, and SPT were conducted. **b** NS shRNA or IL-1β shRNA were well expressed in the HEK293 cells and at the hippocampal microinjection sites in the DG regions of the hippocampus, as indicated by GFP (green) under fluorescence microscopy. Scale bars = 100 μm (HEK293 cell) or 200 μm (hippocampus). **c**–**f** The expressions of GFP (**c**, **d**) and IL-1β (**e**, **f**) in the DG regions of the hippocampus were shown and normalized by the level of β-actin. The data are expressed as the mean ± S.E.M (*n* = 3 per group for western blotting). *p < 0.05 compared with the NS shRNA group

Small interfering RNAs targeting the mouse IL-1β gene were designed by the Shanghai GeneChem Co., Ltd., China. The optimal sequence of small interfering RNAs against mouse IL-1β (5′-TCAAAGGAAAGAATCTATA-3′) was then cloned into the plasmid pGCL-GFP, which encodes a human immunodeficiency virus (HIV)-derived lentiviral vector containing a multiple cloning site for the insertion of shRNA constructs to be driven by an upstream U6 promoter and a downstream cytomegalovirus promoter-GFP (marker gene) cassette flanked by loxP sites. The NS shRNA was constructed by a similar process (5′-TTCTCCGAACGTGTCACGT-3′). These modified plasmids were further co-transfected into HEK293T cells with lentiviral packaging plasmids to generate an IL-1β shRNA-expressing lentivirus or a control shRNA-expressing lentivirus. HEK293T cells were plated in 6-well plates (6 × 10^5^ cells/well) and cultured for 24 h before the transduction of lentiviral vectors (as shown in Fig. 1b). After 2 days of infection, the medium was replaced and the cells were further incubated until 48 h as required.

### Drugs and treatment

LPS from *Escherichia coli* (0111: B4) was purchased from Sigma-Aldrich (St. Louis, MO, USA). The LPS injections were performed intraperitoneally in a 1 mg/kg injection volume. LPS solutions were prepared in saline (0.9% NaCl), and the control was performed intraperitoneally with saline at 10 ml/kg. In addition, malondialdehyde (MDA), superoxide dismutase (SOD), and tumor necrosis factor (TNF-α) test kits were obtained from Nanjing Jiancheng Bioengineering Institute (Nanjing, China). BDNF ELISA kits were obtained from Wuhan Boster Bioengineering Institute (Wuhan, China).

For stereotactic injection of IL-1β shRNA lentivirus (1 μl/side) or NS shRNA lentivirus (1 μl/side) into the dentate gyrus (DG) regions of the hippocampus, the mice were anesthetized with pentobarbital sodium and placed on a stereotactic apparatus (RWD Life Science, Shenzhen, China). The injection was performed through drilled holes in the skull and was into the DG regions of the hippocampus using the following coordinates: − 2.5 mm posterior, ± 2.5 mm lateral, and − 2.0 mm ventral from the bregma. The injection speed was set at 0.2 μl/min, and the needle was left in place for 5 min following the injection. The mice were allowed to recover for 7 days and were handled every other day to reduce the stress associated with handling at the time of testing. The schedule of drug treatment and test orders was shown in Fig. 1a.

### Open field test (OFT)

The OFT was performed in a 50 × 50 × 39 cm white plexiglass box; the box was divided into four identical squares. Line crossing (four paws placed into a new square) and rearing (with both front paws raised from the floor) were recorded for 5 min in a sound-controlled room. The apparatus was cleaned with 5% ethanol to remove scent clues after each test. The OFT was used to evaluate the locomotor activity in the mice.

### Novel object recognition test (NORT)

The NORT was performed in a 50 × 50 × 39 cm white, plexiglass box. In the first day, the mice were subjected to a training session in which the mice were exposed to two of the same objects, and the exploration time was recorded with two stopwatches. Twenty-four hours later, the mice were placed back in the behavioral chamber, and one of the familiar objects was replaced by a novel object. The times spent exploring the familiar and the novel objects were recorded. The NORT was used to evaluate the spatial cognitive ability of the mice.

### Elevated zero maze (EZM)

The EZM was a ring-shaped apparatus, elevated 40 cm from the floor. This apparatus consisted of a circular platform (outer diameter 46 cm, width 5.5 cm) divided into four quadrants of equal lengths with two open arms (with a 1-cm-high curb to prevent falls) and two equal closed arms (surrounded by a 20-cm wall from the surface of the maze). Each mouse was placed at the open arm of the EZM; 5 min of free exploration was recorded by a video camera, and the time spent in the open arm and total number of entries into the open arm during the test was measured, evaluated, and presented. EZM was used to evaluate the anxiety-like behavior in the mice.

### Forced swimming test (FST)

The FST were performed in a glass cylindrical tank that was 60 cm high and 38 cm wide and was filled with fresh water (23 ± 2 °C) to the depth of 40 cm. The total duration of immobility was recorded during the last 4 min of a 6-min time frame. The duration of immobility was considered as the time when the mouse made only the small movements necessary to keep the head above water. The FST was used to evaluate the depression-like behavior in the mice.

### Sucrose preference test (SPT)

The mice were habituated to 1% sucrose for 24 h, and the bottle size was consistent across animals. On testing day, the mice were water-deprived for 6 h and then presented with pre-weighed identical bottles of 1% sucrose and water. The bottles were removed 12 h later and weighed to determine the consumption of each fluid. SPT also evaluated the depression-like behavior in the mice.

### Enzyme-linked immunosorbent assay (ELISA)

Concentrations of MDA, SOD, TNF-α, and BDNF were quantified using ELISA kits according to the manufacturer’s protocol. Hippocampal tissues were homogenized in PBS (Solarbio, Beijing, China) at a ratio of 1:9 (weight to volume) in a glass homogenizer. Supernatant protein concentrations were determined after centrifugation at 12,000 rpm for 10 min at 4 °C. For each sample, 200 μl of extracted protein was used for detection. The absorbance was read on a spectrophotometer at a wavelength of 450 nm. The concentrations of each parameter were calculated according to the standard curve.

### Immunoblotting

Briefly, hippocampal tissues were homogenized in a radioimmunoprecipitation assay (RIPA) lysis buffer (Beyotime, Beijing, China) containing protease inhibitors (Promega, Madison, USA) and phosphatase inhibitors (Sangon, Shanghai, China) and then centrifuged at 12,000 rpm for 30 min at 4 °C. The supernatant was harvested and quantified by the BCA Protein Assay Kit (Beyotime, Beijing, China). Samples (20 μg of protein in each lane) were separated using 10% SDS-PAGE gels and subsequently transferred to PVDF membranes (0.22 μm; Millipore, Temecula, CA, USA). The samples were then incubated overnight with anti-IL-1β (1:1000; Cell Signaling, Danvers, MA, USA), anti-nuclear factor erythroid-derived 2-like 2 (Nrf2) (1:1000; Abcam, Cambridge, MA, USA), rabbit anti-heme oxygenase 1 (HO1) (1:1000; Millipore, Temecula, CA, USA), or rabbit anti-VGF (1:1000; Abcam, Cambridge, MA, USA) primary antibody at 4 °C. Afterward, the membranes were incubated with horseradish peroxidase-conjugated goat anti-β-actin secondary antibodies (1:1000; Cell Signaling Technology, MA, USA) for 60 min. The detection and quantification of specific bands were performed using ImageJ software (NIH, Bethesda, MD, USA).

### Immunohistochemistry

The mice were perfused with 60 ml of saline and 30 ml of 4% paraformaldehyde (PFA). The brain was taken out and post-fixed with same fixative overnight, and then, the brain was treated with 30% sucrose at 4 °C for 2 days. Brain tissue was sectioned with a thickness of 20 μm and pasted into microscope slides. Brain sections were blocked with blocking buffer (BSA, PBS with 0.2% Triton X-100) for 1 h. The sections were incubated overnight with anti-VGF primary antibody or anti-IL-1β primary antibody at 4 °C. The sections were washed in PBS and incubated with fluorescent secondary antibodies, the donkey anti-rabbit-conjugated AlexaFluor 594-labeled and goat anti-rabbit secondary antibodies (1:1000; Invitrogen), for 1 h at room temperature. DNA (nuclei) was stained with DAPI (Solarbio, Beijing, China) for 15 min, mounted onto slides and coverslips with ProLong mounting medium (Invitrogen). The images were captured using the Olympus system and analyzed with ImageJ software.

### Data analysis

Data are expressed as the mean ± standard error of the mean (S.E.M.). Data were analyzed by a two-way ANOVA followed by Newman-Keuls post hoc test using the GraphPad Prism software (Version 6.0, Prism software for PC, GraphPad). The criterion for significance was *p* < 0.05.

## Results

### Inhibition of IL-1β activity produced by IL-1β shRNA blocked the cognitive dysfunction and the anxiety- and depression-like behaviors induced by LPS in mice

NS shRNA or IL-1β shRNA were well expressed in HEK293 cells and at the hippocampal microinjection sites in the DG regions, as indicated by GFP (green) under fluorescence microscopy (as shown in Fig. 1b). The expression of GFP in the DG regions of the hippocampus did not show significant differences between the NS shRNA and IL-1β shRNA-treated mice (*p* > 0.05, Fig. 1c, d). However, IL-1β shRNA lentivirus microinjection into the DG regions of the hippocampus significantly decreased the expression of IL-1β in the DG regions of the hippocampus compared with the NS shRNA lentivirus treatment, indicating the effectiveness of this lentivirus (as shown in Fig. 1e, f).

**Fig. 2:**
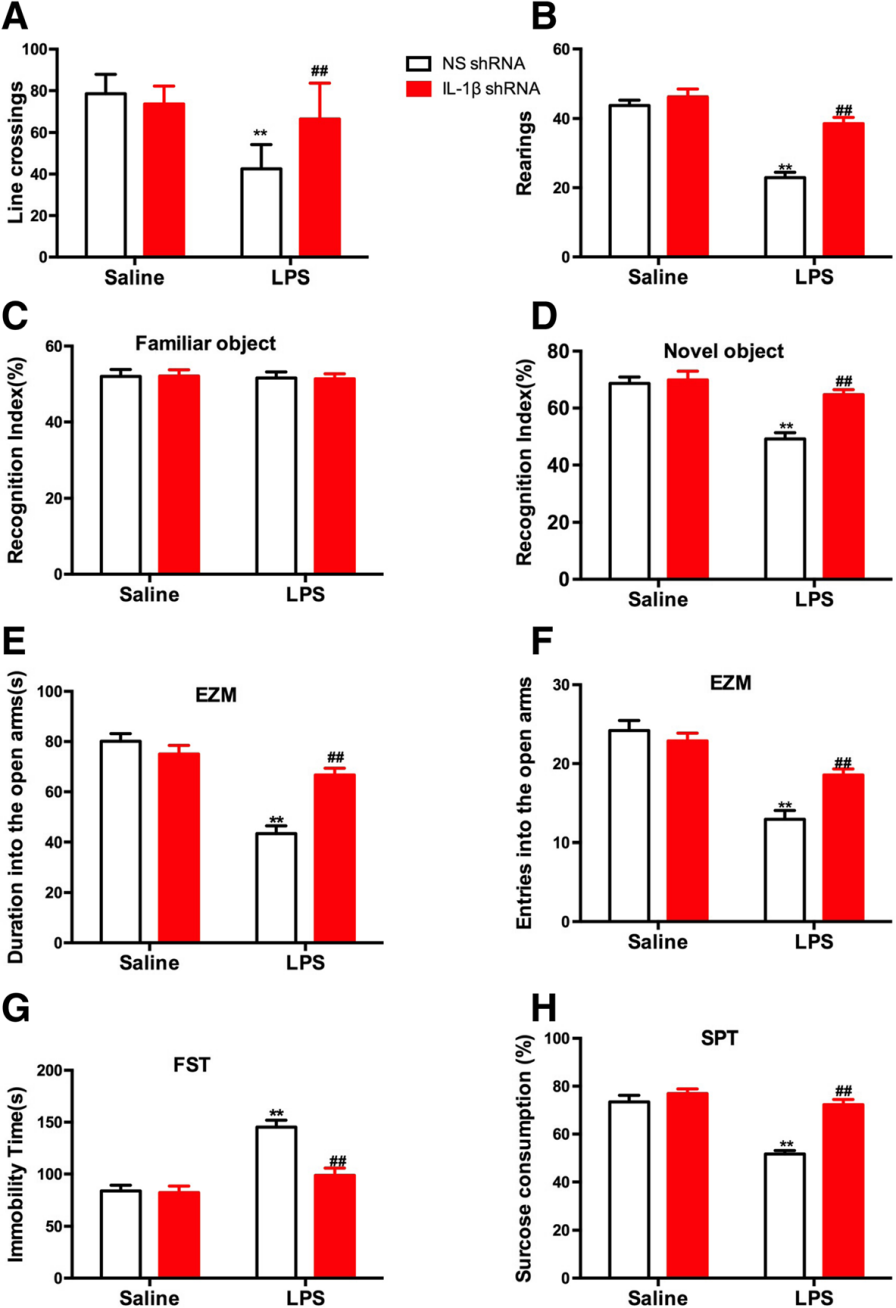
The influence of IL-1β knock-down in the DG regions of the hippocampus on locomotor activity and on anxiety- and depression-like behaviors induced by LPS in mice. Knock-down of IL-1β in the DG regions of the hippocampus alleviated the downregulation of locomotor activity induced by LPS, reflected by the line crossing (**a**) and rearing (**b**) in mice. **c** The recognition index had no significant difference for familiar objects among all the treatments in mice. **d** However, knock-down of IL-1β in the hippocampus alleviated the downregulation in the recognition index for novel objects induced by LPS in mice. The decrease **e** in duration in the open arms and **f** in entries into the open arms induced by LPS was blocked by pretreatment with the IL-1β shRNA lentivirus in the DG regions of the hippocampus of mice. **g** The increase in immobility time in the FST induced by LPS was significantly alleviated by pretreatment with the IL-1β shRNA lentivirus into the DG regions of the hippocampus of mice. **h** The decrease of sucrose consumption in the SPT induced by LPS was blocked by pretreatment with the IL-1β shRNA lentivirus into the DG regions of the hippocampus of mice. The data are expressed as the mean ± S.E.M (*n* = 16 per group). **p < 0.01 compared with the NS shRNA plus saline group; ##*p* < 0.01 compared with NS shRNA + LPS group

To investigate the role of IL-1β in cognitive dysfunction and in the anxiety- and depression-like behaviors induced by LPS in mice, the mice were treated with either IL-1β shRNA lentivirus or NS shRNA lentivirus by microinjection into the DG regions of the hippocampus. After 7 days of recovery, LPS (1 mg/kg, i.p.) or saline were administered. Then, the OFT (Fig. 2a, b) was conducted 24 h after the injection of LPS. The two-way ANOVA revealed significant differences for the LPS treatment [line crossings: *F* (1, 60) = 51.16; *p* < 0.0001; rearings: *F* (1, 60) = 62.08; *p* < 0.0001], the IL-1β shRNA treatment [line crossings: *F* (1, 60) = 10.08; *p* = 0.0024; rearings: *F* (1, 60) = 24.76; *p* < 0.0001] and the LPS treatment × IL-1β shRNA interaction [line crossings: *F* (1, 60) = 22.48; *p* < 0.0001; rearings: *F* (1, 60) = 13.17; *p* = 0.0006] on line crossings (Fig. 2a) and rearings (Fig. 2b). Post hoc analysis showed that the decrease in line crossings (*p* < 0.01) and rearings (*p* < 0.01) produced by LPS was completely prevented by treating the animals with the IL-1β shRNA (both *p* < 0.01).

Additionally, the changes in cognitive ability in the mice were evaluated by NORT. Figure 2c showed that the administration of LPS alone or in combination with IL-1β shRNA did not modify the recognition index for familiar objects of mice in the training session of NORT (LPS treatment [*F* (1, 60) = 0.1210; *p* = 0.7292], IL-1β shRNA treatment [*F* (1, 60) = 0.0018; *p* = 0.9664], LPS treatment × IL-1β shRNA interaction [*F* (1, 60) = 0.0070; *p* = 0.9336]). However, the recognition index for novel objects was significantly changed by LPS alone or in combination with IL-1β shRNA treatment in mice (LPS treatment [*F* (1, 60) = 26.43; *p* < 0.0001], IL-1β shRNA treatment [*F* (1, 60) = 12.17; *p* = 0.0009], LPS treatment × IL-1β shRNA interaction [*F* (1, 60) = 8.906; *p* = 0.0041]). Post hoc analysis shown that the decrease in recognition index (*p* < 0.01) produced by LPS was completely prevented by treating the animals with the IL-1β shRNA lentivirus (*p* < 0.01).

The anxiety-like behaviors were examined by EZM in mice. As shown in Fig. 2e, f, the duration in the open arms (LPS treatment [*F* (1, 60) = 53.29; *p* < 0.0001], IL-1β shRNA treatment [*F* (1, 60) = 8.717; *p* = 0.0045], LPS treatment × IL-1β shRNA interaction [*F* (1, 60) = 21.29; *p* < 0.0001]) and entries into the open arms (LPS treatment [*F* (1, 60) = 54.68; *p* < 0.0001], IL-1β shRNA treatment [*F* (1, 60) = 4.199; *p* = 0.0448], LPS treatment × IL-1β shRNA interaction [*F* (1, 60) = 10.87; *p* = 0.0016]) were significantly changed by LPS and/or IL-1β shRNA treatment in mice. Post hoc analysis showed that the decreases in duration in the open arms (*p* < 0.01) and in entries into the open arms (*p* < 0.01) produced by LPS were completely prevented by treating the animals with the IL-1β shRNA lentivirus (*p* < 0.01 and *p* < 0.01, respectively). Moreover, we also used FST and SPT to demonstrate the depression-like behaviors produced by the LPS alone or in combination with the IL-1β shRNA in mice. As shown in Fig. 2g, h, the immobility time and sucrose consumption were significantly changed by LPS alone [immobility: *F* (1, 60) = 36.37; *p* < 0.0001, Fig. 2g; sucrose consumption: *F* (1, 60) = 38.17; *p* < 0.0001, Fig. 2h], IL-1β shRNA [immobility: *F* (1, 60) = 13.75; *p* = 0.0005, Fig. 2g; sucrose consumption: *F* (1, 60) = 31.52; *p* < 0.0001, Fig, 2h], or in combination with IL-1β shRNA [immobility: *F* (1, 60) = 12.03; *p* = 0.0010, Fig. 2g; sucrose consumption: *F* (1, 60) = 16.08; *p* = 0.0002, Fig. 2h] in mice. Post hoc analysis showed that the increase in immobility time (*p* < 0.01) and the decrease in sucrose consumption (*p* < 0.05) produced by LPS were completely prevented by treating the animals with the IL-1β shRNA lentivirus (*p* < 0.01 and *p* < 0.01, respectively) in mice.

### Inhibition of IL-1β activity mediated by IL-1β shRNA lentivirus alleviated the LPS-induced oxidative response in the hippocampus of mice

**Fig. 3:**
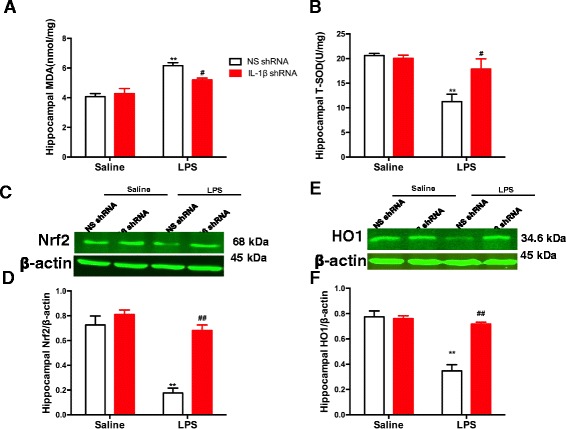
The influence of pretreatment with IL-1β shRNA lentivirus on the levels of oxidative or anti-oxidative parameters induced by LPS in the hippocampus of mice. **a** The upregulation of MDA in the hippocampus induced by LPS was significantly alleviated by IL-1β knock-down in the mice. **b** The downregulation of SOD in the hippocampus induced by LPS was significantly alleviated by IL-1β knock-down in the mice. **c**, **e** Representative immunoblots of Nrf2 and HO1 detected by western blotting with tissues from the hippocampus, and the rest of the panels are quantifications of the immunoblot bands of Nrf2 (**d**) and HO1 (**f**). The decrease in Nrf2 and HO1 in the hippocampus induced by LPS was significantly alleviated by IL-1β knock-down. The data are expressed as the mean ± S.E.M (*n* = 3 per group for western blotting and *n* = 6 per group for ELISA). **p < 0.01 compared with the NS shRNA plus saline group; #*p* < 0.05 or ##*p* < 0.01 compared with the NS shRNA + LPS group

As shown in Fig. 3, the two-way ANOVA revealed significant effects of the LPS treatment [MDA: *F* (1, 20) = 41.86, *p* < 0.0001, Fig. 3a; SOD: *F* (1, 20) = 18.85, *p* = 0.0003, Fig. 3b; Nrf2: *F* (1, 16) = 45.68, *p* < 0.0001, Fig. 3c, d; and HO1: *F* (1, 16) = 41.70, *p* < 0.0001, Fig. 3e, f], the IL-1β shRNA treatment [MDA: *F* (1, 20) = 2.683, *p* = 0.0356; SOD: *F* (1, 20) = 5.151, *p* = 0.0344; Nrf2: *F* (1, 16) = 34.46, *p* < 0.0001; and HO1: *F* (1, 16) = 23.96, *p* = 0.0002], and the LPS treatment × IL-1β shRNA interaction [MDA: *F* (1, 20) = 6.352, *p* = 0.0203; SOD: *F* (1, 20) = 7.235, *p* = 0.0141; Nrf2: *F* (1, 16) = 17.39, *p* = 0.0007; and HO1: *F* (1, 16) = 27.83, *p* < 0.0001] on the levels of MDA, SOD, Nrf2, and HO1 in the hippocampus of mice. Post hoc analysis showed that the upregulation of MDA (*p* < 0.01, Fig. 3a) and the downregulation of SOD (*p* < 0.05), Nrf2 (*p* < 0.01), and HO1 (*p* < 0.01) induced by LPS were completely prevented by treating the animals with the IL-1β shRNA lentivirus in the hippocampus.

### Inhibition of IL-1β activity by IL-1β shRNA blocked LPS-induced pro-inflammatory cytokines in the hippocampus of mice

**Fig. 4:**
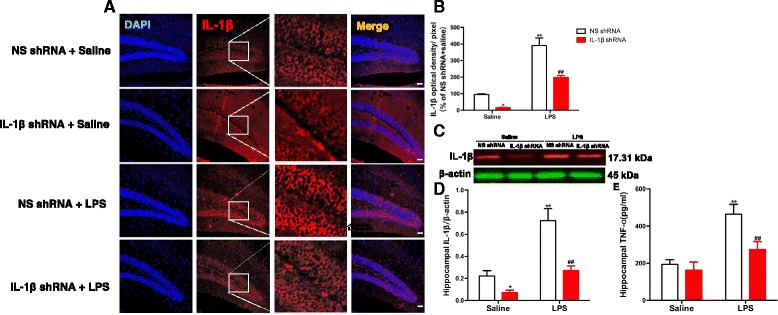
The upregulation of neuroinflammatory factors in the hippocampus induced by LPS was blocked by pretreatment with IL-1β shRNA lentivirus in mice. **a** Mouse hippocampus samples were examined by using immunofluorescent analysis; sections were labeled with anti-IL-1β antibody (red) and with fluorescent nuclear DAPI staining (blue) and visualized using a fluorescence microscope. Scale bar = 50 μm. **b** Quantification of the IL-1β optical density per pixel was significantly decreased by IL-1β shRNA lentivirus and showed that the upregulation of IL-1β levels induced by LPS was significantly alleviated by the IL-1β shRNA lentivirus in mice. **c**, **d** The hippocampal IL-1β protein expression was significantly decreased by LPS, and the IL-1β shRNA lentivirus alleviated this effect. **e** The upregulation of TNF-α in the hippocampus induced by LPS was significantly alleviated by pretreatment with IL-1β shRNA lentivirus in mice. The data are expressed as the mean ± S.E.M (*n* = 3 per group for western blotting and *n* = 6 per group for immunofluorescent analysis or ELISA). *p < 0.05 or **p < 0.01 compared with the NS shRNA plus saline group; ##*p* < 0.01 compared with the NS shRNA + LPS group

As shown in Fig. 4a, b, the IL-1β-labeled positive cells in the DG regions of the hippocampus were significantly changed by the LPS treatment [*F* (1, 20) = 99.18; *p* < 0.0001], IL-1β shRNA treatment [*F* (1, 20) = 32.65; *p* < 0.0001] and the LPS treatment × IL-1β shRNA interaction [*F* (1, 20) = 5.717; *p* = 0.0267]. Post hoc analysis showed that the increase of IL-1β (*p* < 0.01, Fig. 4b) induced by LPS was completely prevented by treating the animals with the IL-1β shRNA (*p* < 0.01). Additionally, the protein expression of IL-1β in the DG regions of the hippocampus was significantly changed by the LPS treatment [*F* (1, 16) = 149.6; *p* < 0.0001], the IL-1β shRNA treatment [*F* (1, 16) = 110.1; *p* < 0.0001], and the LPS treatment × IL-1β shRNA interaction [*F* (1, 16) = 27.16; *p* < 0.0001]. Post hoc analysis showed that the increase in IL-1β (*p* < 0.01, Fig. 4c, d) induced by LPS was completely prevented by treating the animals with the IL-1β shRNA (*p* < 0.01). Furthermore, the level of TNF-α in the hippocampus was significantly changed by the LPS treatment [*F* (1, 20) = 121.1; *p* < 0.0001], the IL-1β shRNA treatment [*F* (1, 20) = 41.03; *p* < 0.0001], and the LPS treatment × IL-1β shRNA interaction [*F* (1, 20) = 20.91; *p* = 0.0002]. Post hoc analysis showed that the increase in TNF-α (*p* < 0.01, Fig. 4e) induced by LPS was completely prevented by treating the animals with the IL-1β-shRNA lentivirus.

### Inhibition of IL-1β activity by IL-1β shRNA lentivirus blocked the downregulation of VGF and BDNF by LPS in the hippocampus of mice

**Fig. 5:**
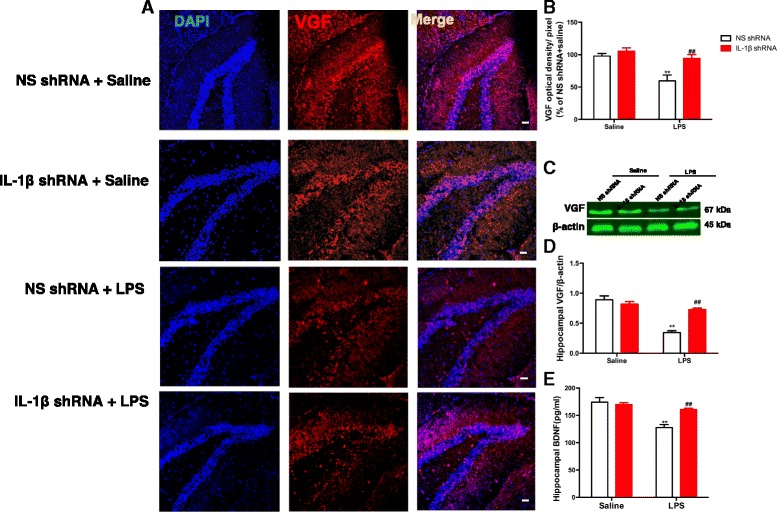
The downregulation of VGF and BDNF in the hippocampus induced by LPS was alleviated by pretreatment with IL-1β shRNA lentivirus in mice. **a** Mouse hippocampus samples were examined by using immunofluorescent analysis; sections were labeled with the anti-VGF antibody (red) and with fluorescent nuclear DAPI staining (blue) and visualized using a fluorescence microscope. Scale bar = 50 μm. **b** Quantification of the VGF optical density per pixel was significantly decreased by LPS and was significantly alleviated by pretreatment with the IL-1β shRNA lentivirus in mice (*n* = 3 per group). **c** Representative immunoblots of VGF detected by western blotting with tissues from the hippocampus, and the rest panel is quantification of the immunoblotting bands of VGF (**d**). The downregulation on VGF in the hippocampus induced by LPS was significantly blocked by pretreatment with the IL-1β shRNA lentivirus in mice. **e** The downregulation of BDNF in the hippocampus induced by LPS was significantly blocked by pretreatment with the IL-1β shRNA lentivirus in mice. The data are expressed as the mean ± SEM (*n* = 3 per group for western blotting and *n* = 6 per group for ELISA). **p < 0.01 compared with the NS shRNA plus saline group; ##*p* < 0.01 compared with the NS shRNA + LPS group

Finally, we examined the expressions of VGF (Fig. 5a–d) and BDNF (Fig. 5e) in the hippocampus of mice. The fluorescent expression (Fig. 5a, b) and protein expression (Fig. 5c, d) of VGF were significantly changed by the LPS treatment [fluorescent expression: *F* (1, 20) = 14.66; *p* = 0.0010; protein expression: *F* (1, 16) = 55.21; *p* < 0.0001], the IL-1β shRNA treatment [fluorescent expression: *F* (1, 20) = 10.57; *p* = 0.0040; protein expression: *F* (1, 16) = 13.00; *p* = 0.0024], and the LPS treatment × IL-1β shRNA interaction [fluorescent expression: *F* (1, 20) = 4.493; *p* = 0.0467; protein expression *F* (1, 16) = 27.84; *p* < 0.0001]. However, pretreatment with the IL-1β shRNA lentivirus significantly alleviated the downregulating effects of LPS (fluorescent expression: *p* < 0.01; protein expression: *p* < 0.01). In addition, as shown in Fig. 5e, the two-way ANOVA revealed significant effects of the LPS treatment [*F* (1, 20) = 27.04; *p* < 0.0001], the IL-1β shRNA treatment [*F* (1, 20) = 7.076; *p* = 0.0150], and the LPS treatment × IL-1β shRNA interaction [*F* (1, 20) = 12.26; *p* = 0.0022] on the levels of BDNF in the hippocampus of mice. However, the knock-down of IL-1β in the DG regions of the hippocampus significantly blocked the effects of LPS (*p* < 0.01).

## Discussion

A growing body of evidence suggests that activation of the immune response following systemic infection often results in neuroinflammatory responses and consequently induces neuropsychiatric symptoms in animal models and humans [7–10, 24–26]. Specifically, inflammation in the context of the nervous system, termed “neuroinflammation,” has been reported in patients with neuropsychiatric disorders and is typically associated with microglial activation and the production of cytokines. Pro-inflammatory cytokines, including IL-1β and TNF-α, are secreted primarily by the microglia [27–29]. A recent study also raised the possibility that cytokines might be used as endogenous biomarkers of the efficacy of antidepressants [30]. Notably, IL-1β has been proposed to be a key mediator in a variety of behavioral actions of neuropsychiatric disorders [31]. Once activated, IL-1β can activate immune-related cells, such as monocytes, to produce the corresponding immune effects, which in turn aggravate the inflammatory reaction [32]. Moreover, pharmacological inhibition of IL-1β signaling has been shown to be beneficial in some autoimmune and autoinflammatory diseases, making IL-1β a promising therapeutic target in neuroinflammatory conditions [11, 12, 33]. The first objective of our present study was to explore the involvement of IL-1β in the behavioral dysfunction induced by a peripheral immune challenge in mice. The mechanisms of central or peripheral treatment with LPS on behavioral dysfunctions in rodents trigger an increase in the expression of pro-inflammatory cytokines [7, 34, 35]. Herein, we found that peripheral administration of LPS causes the elevation of IL-1β and TNF-α levels in the hippocampus of mice, which may have contributed to the behavioral alterations. Additionally, our current data are consistent with previous evidence that indicates that neuroinflammation may play a crucial role in the pathophysiology of anxiety, major depression, and recognition memory deficits [36–38]. Interestingly, our present study investigated the significant alleviating effects of IL-1β knock-down in the DG regions of the hippocampus on the memory deficits and the depression- and anxiety-like behaviors, indicating the key role of IL-1β in the neuropsychiatric dysfunctions induced by LPS in mice.

In addition to the neuroinflammation induced by LPS administration in rodents, oxidative stress may also play an important role in neuropsychiatric disorders. Our current observations further confirmed that the levels of markers reflecting the status of oxidative stress, such as MDA, were significantly altered by LPS administration. However, reduced SOD, Nrf2, and HO1 levels by LPS were also found in our present study, indicating that reversion of the harmful changes in oxidative stress markers would be beneficial for neuroinflammation-induced neuropsychiatric disorder therapy. Nrf2, a transcription factor involved in the cellular defense against oxidative stress and the neuroinflammatory response, is one potential target of interest for the treatment of neuropsychiatric disorders [39–41]. In addition, the cytoprotective enzyme HO1 is an Nrf2 target protein and HO1 upregulation contributes to the antioxidant and anti-inflammatory properties [42, 43]. However, very few studies have assessed whether SOD, Nrf2, and HO1 were downregulated by IL-1β in the neuropsychiatric dysfunctions induced by LPS in mice. Herein, one of the most striking findings of our current study was that IL-1β knock-down in the hippocampus of mice significantly antagonized the LPS-induced decrease in SOD, Nrf2, and HO1. Thus, neuroinflammation, along with oxidative stress, perpetuates a vicious cycle that ultimately leads to the behavioral alterations associated with neuropsychiatric disorders.

BDNF and VGF are involved in many aspects of neuronal functioning including synaptic plasticity, neurogenesis, and neuronal survival and are associated with core features of neuropsychiatric disorders [17, 44–46]. Concerning our results, LPS administration was associated with decreased BDNF and VGF levels in the brains of mice, which is in agreement with previous studies that have found that cytokines were increased in neuropsychiatric patients [36], which then led to neurotoxic effects by pro-inflammatory cytokines and oxidative stress-associated mechanisms that ultimately impaired BDNF and VGF expression [7, 47, 48]. Specifically, our data show that IL-1β knock-down alleviates the downregulation of BDNF and VGF in the hippocampus of LPS-treated mice. Our current data may further demonstrate the key role of IL-1β in the regulation of BDNF and VGF after LPS treatment in mice. Our results are consistent with a recent report that demonstrated that the pharmacological inhibition of IL-1β prevented depression-like behaviors [49], indicating that IL-1β is a key component of the inflammatory response of the brain and mediates the effect of inflammation on depression-like behaviors in mice. However, IL-1β, acting via poorly understood mechanisms, appears to be a key cytokine in causing decreases in BDNF and VGF along with presumably related behavioral dysfunctions induced by LPS in mice. Recent studies demonstrated that IL-1β suppressed TrkB-mediated BDNF signaling and the cAMP response element-binding protein (CREB) [50], a transcription factor regulating BDNF and VGF expression [51, 52]. These findings may explain the mechanism of the involvement of IL-1β in the LPS-induced downregulation of BDNF and VGF in mice, suggest that IL-1β is critical for the development of depression-like behaviors, and highlight IL-1β as a potential novel therapeutic target for the treatment of mood disorders.

## Conclusion

Taken together, alterations in the pro-inflammatory cytokines and oxidative stress parameters may contribute to the molecular basis of memory deficits and anxiety- and depression-like behaviors. To our knowledge, our present study is the first to report that IL-1β knock-down protects neuroinflammatory and oxidative responses, leading to VGF and BDNF activation in the hippocampus of mice. Our current study may provide novel insights into the development of new therapeutic approaches for neuropsychiatric disorders.

## Abbreviations

BDNF: Brain-derived neurotrophic factor
CREB: cAMP response element-binding protein
DG: Dentate gyrus
ELISA: Enzyme-linked immunosorbent assay
EZM: Elevated zero maze
FST: Forced swimming test
HO1: Heme oxygenase 1
ICR: Imprinting control region
IL-1β: Interleukin-1β
LPS: Lipopolysaccharide
MDA: Malondialdehyde
NORT: Novel object recognition test
Nrf2: Nuclear factor erythroid-derived 2-like 2
OFT: Open field test
RIPA: Radioimmunoprecipitation assay
SOD: Superoxide dismutase
SPT: Sucrose preference test
TNF-α: Tumor necrosis factor-α
